# Chondroitin Sulphate/Dermatan Sulphate Proteoglycans: Potential Regulators of Corneal Stem/Progenitor Cell Phenotype In Vitro

**DOI:** 10.3390/ijms24032095

**Published:** 2023-01-20

**Authors:** Kiranjit K. Bains, Sean Ashworth, Elena Koudouna, Robert D. Young, Clare E. Hughes, Andrew J. Quantock

**Affiliations:** 1 Structural Biophysics Group, School of Optometry and Vision Sciences, Cardiff University, Maindy Road, Cardiff CF24 4HQ, UK; 2 School of Biosciences, College of Biomedical and Life Sciences, Cardiff University, Cardiff CF10 3AX, UK

**Keywords:** cornea, chondroitin sulphate, dermatan sulphate, keratocytes, glycosaminoglycans, stem cell niche

## Abstract

Chondroitin sulphate (CS) proteoglycans with variable sulphation-motifs along their glycosaminoglycan (GAG) chains are closely associated with the stem cell niche of articular cartilage, where they are believed to influence the characteristics of the resident stem cells. Here, we investigated the immunohistochemical distribution of hybrid CS/dermatan sulphate (DS) GAGs in the periphery of the adult chicken cornea, which is the location of the cornea’s stem cell niche in a number of species, using a monoclonal antibody, 6C3, that recognises a sulphation motif-specific CS/DS GAG epitope. This revealed positive labelling that was restricted to the subepithelial corneal stroma, as well as nearby bony structures within the sclera, called ossicles. When cultivated on cell culture dishes coated with 6C3-rich CS/DS, corneal stromal cells (keratocytes) that had been isolated from embryonic chicken corneas formed circular colonies, which took several days to reach confluency. A flow cytometric analysis of these keratocytes revealed changes in their expression levels of the indicative stem cell markers, Connexin 43 (Cx43), Paired Box 6 (*PAX6*), B-lymphoma Moloney murine leukemia virus insertion region-1 (Bmi-1), and C-X-C Chemokine Receptor 4 (CXCR4) suggestive of a less-differentiated phenotype compared with expression levels in cells not exposed to CS/DS. These findings support the view that CS/DS promotes the retention of a stem cell phenotype in corneal cells, much as it has been proposed to do in other connective tissues.

## 1. Introduction

Proteoglycans (PGs) are extracellular matrix components that consist of a protein core covalently bonded to one or more glycosaminoglycan (GAG) chains. In the corneal stroma, PGs with hybrid chondroitin sulphate/dermatan sulphate (CS/DS) GAGs have been shown to contribute to collagen fibrillogenesis and organisation, in addition to regulating a spectrum of signalling molecules during corneal development [1,2,3,4,5]. Structurally, the CS chain is a repeating disaccharide sequence of alternating glucuronic acid and N-acetyl-galactosamine residues and, through the epimerisation of glucuronic acid to iduronic acid, CS is converted to DS [6,7]. Modifications to the sulphation of CS/DS chains at the C-4 and/or C-6 position of N-acetyl-galactosamine residues and/or the C-2 position of glucuronic acid/iduronic acid residues [8,9,10,11,12] have the potential to generate many different variants, estimated at over one thousand per small pentasaccharide unit [13]. This affects the physical and chemical properties as well as the biological and pharmacological activity of CS/DS molecules within their respective environments [11,12,14]. The highly specific manner in which these sulphation motifs are thought to interact with other matrix molecules, including growth factors, cytokines and chemokines, directly influences various cascades of events involved in cell proliferation, differentiation, migration and matrix secretion [15].

CS/DS is also associated with maintaining embryonic stem cell pluripotency in early embryogenesis. Investigations with knockout mice, for example, found that in the absence of CS, embryonic cell division was inhibited before the eight-cell stage due to the failure of cytokinesis [15]. Subsequent studies utilizing glucuronyltransferase-I-knockout embryonic stem cells revealed that CS is a novel determinant in controlling the functional integrity of embryonic stem cells via binding to E-cadherin [16]. Similarly, others have reported that the enzymatic elimination of endogenous CS/DS with chondroitinase ABC affected the expression of pluripotency markers by embryonic stem cells, leading to defects in cardiac lineage determination [17]. Parallel effects on the ability to maintain pluripotency were also observed in neuronal stem cells, with Sirko and associates [18] discovering that removing CS/DS from culture prevented neurosphere formation. This implies that CS/DS has a necessary role in neurogenesis and maintenance of the neural stem/progenitor cell niche.

Of course, CS/DS is not the only GAG associated with stem cell behaviour, and numerous studies have shown that heparan sulphate (HS) is closely associated with the development of several tissues [19,20,21,22]. In particular, HS is known to control the mouse embryonic stem cell fate because it is required for exit from self-renewal [23]. With regards to the cornea, it is also noteworthy that extracellular sulfatases for HS promote the migration of corneal epithelial cells during wound repair in mice [24] and that the conditional knockout of murine HS perturbs the phenotype of corneal epithelial cells, though not keratocytes, and delays corneal epithelial wound healing [25]. Hyaluronic acid (hyaluronan), moreover, similar to CS/DS, is also a component of the corneal limbal stem cell niche where it is believed to help maintain the limbal epithelial stem cell phenotype [26]. Similar to the current work with CS/DS, hyaluronan has been used in recent studies as a substrate to support limbal epithelial stem cell growth in culture [27], as well as being fairly widely used in clinical trials, in cartilage mostly, to improve the survival and retention of injected stem cells for tissue regeneration, or to guide endogenous stem cells towards a specific site or injured area [28].

In the early 1980s, Caterson and colleagues were the first to generate and characterise monoclonal antibodies that recognise specific sequences of native and non-native disaccharide/oligosaccharide sulphation motif epitopes domains within the framework of CS/DS chains [8,13,29,30,31]. For example, it was determined that antibody 6C3 recognised native epitopes occurring towards the non-reducing terminus of CS/DS chains [8]. Subsequently, 6C3 and other antibodies have served as the foundation for decades of study into identifying specific CS/DS motifs occurring spatio-temporally in tissue and organ development [13,31]. More recently, they have been used to investigate the role of CS/DS with respect to the stem/progenitor niche in various organ systems [13,29,32].

Investigations into the mechanism of cartilage degradation, characteristic of osteoarthritis, for example, have shown the articular cartilage surface to be a location for the regulation of morphogenesis and growth via the synthesis of differential matrix components [33], in addition to being a signalling centre as shown through the expression of numerous growth factors and localisation of specific cell receptors [34,35,36]. Hayes and associates have also reported clear labelling with several specific anti-CS monoclonal antibodies (i.e., 4C3, 7D4 and 3B3[−]) within a distinct microenvironment of the superficial zone of cartilage, previously reported to contain the stem/progenitor cell niche [29]. They went on to propose a hypothetical model in which CS PGs carrying non- and/or lesser-sulphated GAGs act as a physical and biochemical barrier around stem cells, which implies the direct involvement of CS PGs in the maintenance of the stem/progenitor cell niche. The authors further suggested that when cells translocated out of the CS-rich niche daughter cells would become exposed to signalling molecules, leading to differentiation [29].

Although research into osteoarthritis has helped to advance our understanding of a potential role for CS/DS GAGs in maintaining the stem cell niche in articular cartilage, relatively few studies have examined the potential role of these molecules in supporting the presumed corneal stem cells (epithelial and stromal) at the limbus at the edge of the cornea [13,21,29,32]. Previously, we reported a distinct pattern of CS/DS distribution in mature porcine, rabbit and human eyes using anti-CS/DS sulphation motif-specific antibodies, including 6C3 [5,37]. Tissue-specific immunolocalisation was present at the corneal limbus associated with deep limbal vasculature and the extracellular matrix along the basement membrane at the site of limbal epithelial and mesenchymal cell interactions. This is an emerging field with relevance to corneal tissue engineering strategies, and work is still required to clarify the involvement of CS/DS as a potential modulator of the corneal stem cell niche. 

A sub-population of mesenchymal cells, characteristic of adult stem cells and termed corneal stromal stem cells, have been found within the stromal matrix in the region of the limbal stem cell niche. These cells are believed to actively support multiple cell types within the cornea to maintain corneal transparency [38]. Much like corneal stromal stem cells, stromal keratocytes are also derived from mesenchymal cells. With regards to late-stage embryonic keratocytes, data has indicated that they are not terminally differentiated, but instead exist as partially-restricted precursors [39,40,41]. Lwigale and co-workers [39] demonstrated the multipotency of these cells through the introduction of later-stage embryonic keratocytes into various neural crest-derived populations of earlier embryos. Translocated keratocytes successfully proliferated, migrated and dedifferentiated into many of the neural-crest populations indicating that keratocytes were partially restricted progenitor cells. Similar findings were also reported by Chao and associates [42] who used human foetal keratocytes in a similar investigation [39,40,42].

More recently, cultured corneal stromal stem cells isolated from human corneas were used to evaluate the expression of stem cell markers and genes associated with pluripotent cells, mesenchymal stem cells, neural stem/progenitor cells and ocular precursors in embryonic development [38]. Using flow cytometry, the authors were able to isolate adult stem cell sub-populations and quantify the percentage of cells expressing selected stem cell markers. In the present study, we confirm that, as is the case for other species [5,37], 6C3-immunopositive CS/DS (6C3^+^ CS/DS) is present in the limbal region of the chicken corneal stroma. Then, to test the hypothesis that CS/DS influences the multipotency and differentiation propensity of corneal stromal stem/progenitor, keratocytes obtained from 18-day-old embryonic chick corneas (i.e., a few days before hatching) were cultured on clean plastic substrates and on those coated with the CS/DS epitope recognised by the 6C3 antibody. Flow cytometry was subsequently employed to ascertain expression levels of the indicative stem cell markers, Connexin 43 (Cx43), Paired Box 6 (*PAX6*), B-lymphoma Moloney murine leukemia virus insertion region-1 (Bmi-1), and C-X-C Chemokine Receptor 4 (CXCR4), which indicated that a less-differentiated state had been achieved in chick corneal keratocytes cultivated in the presence of 6C3^+^ CS/DS. 

## 2. Results

### 2.1. 6C3^+^ CS/DS Epitope in the Adult Avian Cornea

**Figure 1:**
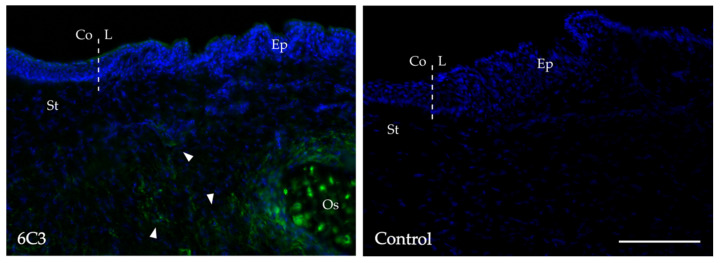
Immunolabelling of chondroitin sulphate (CS) proteoglycans in the adult avian limbus. Positive labelling (green) of CS specific epitopes recognised by monoclonal antibody 6C3 was observed in the subepithelial midstroma (white arrowheads). Nuclei counterstained with DAPI (blue). Dashed vertical white lines represent the corneolimbal junction. The primary antibody was omitted from the control section. Ep = epithelium, St = stroma, L = limbus, Co = cornea. Os = scleral ossicle. Images are representative from three individual experiments. Scale bar represents 100 µm.

Anti-CS/DS antibody 6C3 was applied to adult avian corneoscleral tissue (Figure 1), revealing distinct positive labelling (green) limited to the limbal region, primarily in the corneal stromal matrix. Furthermore, high levels of 6C3 labelling were also visible focally in scleral ossicles, bony structures present in the chicken eye, reflective of the presence of CS bone marrow and musculoskeletal tissues [13]. Negative control sections, with the primary antibody omitted, confirmed a lack of non-specific binding of a secondary antibody. 

### 2.2. 6C3^+^ CS/DS Influences Cell Growth In Vitro 

**Figure 2:**
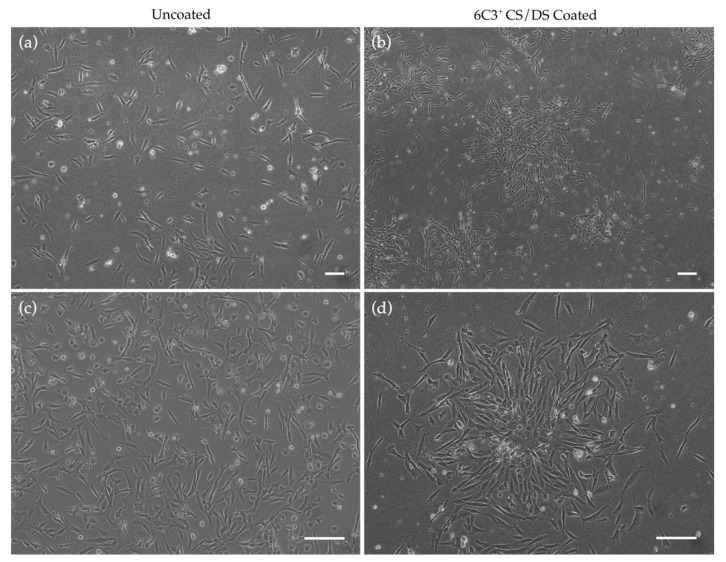
Phase contrast microscopy showing variation in growth pattern of E18 keratocytes cultured on uncoated and CS/DS coated substrate. (**a**,**c**) P0 cells cultured on uncoated dishes show diffuse growth across the dish taking up to two days to reach 100% confluency, whereas (**b**,**d**) cells cultured on 6C3^+^ CS/DS coated culture dishes show spherical colony growth with 100% confluency being reached after seven days. (**a**,**c**) represent different regions of the uncoated dishes taken at different magnifications. (**b**,**d**) represent different regions of the coated dishes taken at different magnifications. Representative images of three independent experiments. Scale bars represents 100 μm.

Keratocytes isolated from chick corneas at day 18 of embryogenesis (E18), a few days before hatching, were seeded at a density of 3.0 × 10^5^ cells/mL on uncoated cell culture plates and on culture plates that had been coated with a CS/DS GAG substrate. The CS/DS GAG chains, obtained from shark skeletal cartilage, were enriched in the CS/DS epitope recognised by the 6C3 antibody and were determined to be free of other GAG species, including keratan sulphate (KS) as recognised by the 5D4 antibody, a common contaminant of purified CS. At passage 0 (P0), cells on uncoated dishes grew in a non-specific, diffuse manner taking an average of two days to reach confluency, whereas cells grown on 6C3^+^ CS/DS coated culture dishes formed multiple, colony-like aggregates across the dish, taking more than seven days on average to reach 100% confluency when seeded at the same density (Figure 2). 

### 2.3. Phenotypic Status of Cells In Vitro

**Figure 3:**
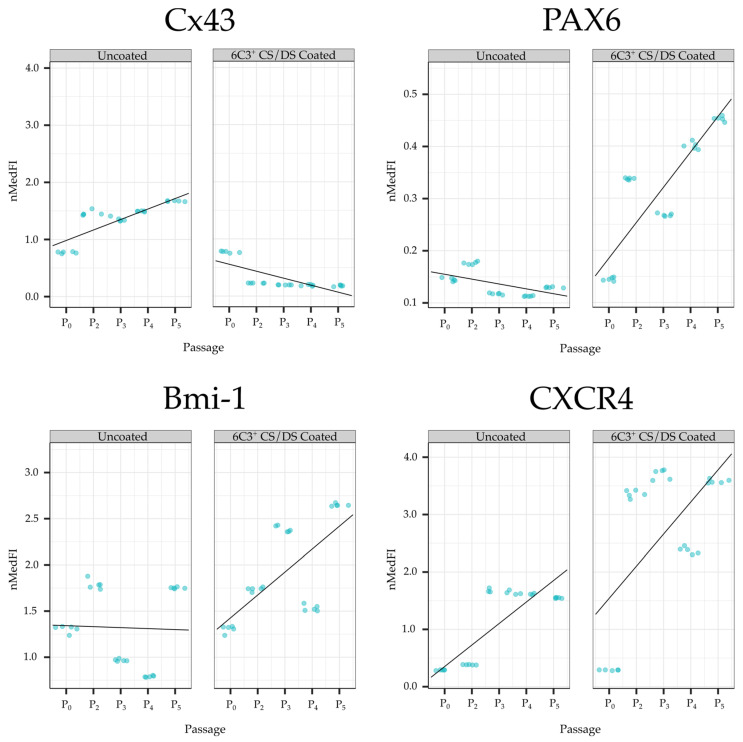
Linear regression showing the relationship between nMedFI and passage number in each coating condition for Cx43, *PAX6*, Bmi-1 and CXCR4. Each point represents a repeated instrument measure of nMedFI at each passage for each PSSC marker and coating.

To ascertain the phenotypic status of cultured keratocytes, an analysis of marker expression (*normalised median fluorescence intensity, nMedFI*) for coated and uncoated culture conditions and passage was conducted (Table 1), with Cx43, *PAX6*, Bmi-1 and CXCR4 chosen as indicative positive or negative markers of stem/progenitor cell status. In addition to this, the average marker expression across all passages for each coating condition was examined (Table 1). Compared to cells grown on the uncoated control surfaces, those grown on 6C3^+^ CS/DS substrates showed a lower average cell expression of Cx43 and a higher average expression for *PAX6*, Bmi-1, and CXCR4. As seen in Table 2, a paired student *t*-test of marker expression obtained for 6C3^+^ CS/DS coated and uncoated conditions was significant (*p* < 0.001). Additional analysis using a linear regression was performed to assess marker expression (*nMedFI*) against passage for both coating conditions (Figure 3), summarised in the form of regression coefficients in Table 3. For Cx43, the regression coefficients reported a significant positive relationship (*p* < 0.001) between nMedFI and passage for the uncoated condition and a significant negative relationship (*p* < 0.001) for the 6C3^+^ CS/DS coating condition. For *PAX6*, the regression coefficients reported a significant negative relationship (*p* < 0.05) for the uncoated condition and a significant positive relationship (*p* < 0.001) for the 6C3^+^ CS/DS coating condition. For Bmi-1, the regression coefficients reported a significant positive relationship (*p* < 0.001) for the 6C3^+^ CS/DS coating only. For CXCR4, the regression coefficients reported a significant positive relationship (*p* < 0.001) for both coating conditions. 

## 3. Discussion

### 3.1. Immunolocalisation in the Cornea of Epitope-Specific CS/DS Antibody 6C3

In recent years, the criteria for confirming stem cell potential have broadened to include the matrix localisation of specific epitopes in the niche microenvironment where cues for the maintenance of stem cell characteristics may reside [43]. One avenue of investigation has seen monoclonal antibodies that recognise sulphation patterns along CS/DS GAG chains used to identify regions of rat intervertebral discs and human foetal elbows that are associated with proliferating chondrocytes [13,29,31,32]. In the cornea, a population of epithelial and stromal stem/progenitor cells is known to reside in the limbal stem cell niche near the junction of the cornea and sclera in a number of species [5,38,43]. Previously, we reported positive labelling for 6C3 in the porcine and rabbit corneoscleral limbus [5,37]. The current observations of 6C3 labelling in the avian limbus (Figure 1) further demonstrates the consistent localisation of 6C3^+^ CS/DS in the limbal region across species, with the antibody identifying GAG epitopes that reside towards the non-reducing terminus of the GAG chain [8,13,44]. 

### 3.2. The Effect of 6C3^+^ CS/DS on Keratocyte Growth In Vitro

In the current study, micrographs were acquired of embryonic keratocytes cultured on 6C3^+^ CS/DS coated and uncoated culture plates. Qualitatively, the findings showed obvious differences in the pattern of cell growth. Notably, when keratocytes were cultured on 6C3^+^ CS/DS substrate, they formed multiple colony-like aggregates across the dish surface despite being seeded as a single cell suspension. On the other hand, when keratocytes were cultured on uncoated control plates, diffuse adherence was observed without aggregate formation. Funderburgh’s group noted that keratocyte-derived fibroblasts lost the ability to form spheroids in vitro and regarded spheroid-formation as a specialised characteristic maintained by cloned progenitor cells [45]. In this case, it can be speculated that cells cultured on 6C3^+^ CS/DS substrate are not adopting a differentiated phenotype but rather a less-differentiated progenitor phenotype.

### 3.3. The Effect of 6C3^+^ CS/DS on the Phenotypic Expression Profile of Keratocytes In Vitro 

Flow cytometric analysis showed a significant difference between the phenotypic profiles of indicative stromal stem cell markers expressed by embryonic keratocytes cultured on 6C3^+^ CS/DS coated and uncoated culture plates. Notably, keratocytes cultured on 6C3^+^ CS/DS revealed an expression profile suggestive of cells adopting a less differentiated, progenitor-like phenotype compared to cells grown in the absence of 6C3^+^ CS/DS. Specifically, keratocytes cultured on the 6C3^+^ CS/DS showed a down-regulation in Cx43 expression combined with an up-regulation of *PAX6*, Bmi-1 and CXCR4 expression when compared to cells grown in uncoated conditions. This finding provides support for the hypothesis proposed by Hayes and associates [29,32] that differentially sulphated CS/DS GAGs play a role in regulating the proliferation and differentiation potential of stem/progenitor cells within the stem cell niche of a number of connective tissues. 

It is widely known that connexins play an integral role in directing cell-cell communications which mediate cellular processes including proliferation, differentiation and apoptosis [46]. Chen and colleagues were amongst the first to assert that Cx43 was a reliable negative marker for stem cell populations, observing that primary limbal cultures harboured subpopulations of Cx43-negative cells, which they characterised as being small, less differentiated and with a propensity for proliferation [46,47,48]. Isolated Cx43-negative cells were later shown to represent, at very early stages in ocular development, precursors of basal and putative stem cells of the limbal epithelium [47,49]. Correspondingly, Matic and co-workers [50,51] demonstrated that stem cells of both corneal epithelium and epidermis also lacked connexins, as did presumptive stem cells within mice vibrissae. Given these findings, the evident down-regulation of Cx43 by keratocytes cultured on enhanced 6C3^+^ CS/DS substrates could be viewed as cells adopting a less-differentiated phenotype. Matic and associates also speculated that the presence of Cx43 would make a cell vulnerable to insult, which in turn would affect neighbouring cells, whereas the absence of Cx43 would confer survival advantageous to multipotent cells [50]. 

*PAX6* is fundamental to early eye development and is expressed by several embryonic ocular tissues, but has been regarded as being absent from the corneal stroma [45,52]. Subsequent reports, however, found that in the adult corneal stroma, a subpopulation of progenitor cells (~4% of the total population) demonstrated intense immunostaining for *PAX6*, in addition to retaining the ability to divide extensively and generate adult keratocytes, unlike primary keratocytes cultured under the same culture conditions [40,45,53]. Progenitor cells have been distinguished from keratocytes on the basis of their up-regulation of Bmi-1, a polycomb complex protein essential in the self-renewal of stem cells in a number of organ systems [45]. The combined up-regulation of *PAX6* and Bmi-1 was found here for cells grown on 6C3^+^ CS/DS, whereas for uncoated conditions Bmi-1 was seen to decline throughout repeated passages. These findings further suggest that cells cultured on CS/DS are assuming a less-differentiated state and, we speculate, may be gaining both multipotency and self-renewal capabilities. 

Interestingly, a number of studies have shown that corneal stromal stem cells support the maintenance of native populations of epithelial stem cells in the limbal niche [54,55,56,57]. These limbal niche cells, which are in close anatomical proximity to basal cells, modulate limbal stem/progenitor cells via the CXCR4/Stromal cell-derived factor-1 (SDF-1) receptor ligand axis [58,59]. This was exemplified by Xie and associates [58] who found that when CXCR4, a surface chemokine receptor which is strongly expressed by human limbal stromal niche cells, was inhibited, it disrupted the consolidation of co-isolated populations in culture. In addition, epithelial spheres exhibited the loss of holoclone formation and greater corneal differentiation [58]. That study provided for the first time strong evidence that limbal stem cell function was linked to the close physical association with native niche cells via the CXCR4/SDF-1 axis [58]. More recent work by Funderburgh and colleagues [38] has also substantiated the high levels of CXCR4 expression in subpopulations of human corneal stromal stem cells. These reports, taken together with our data showing elevated CXCR4 expression in cells cultured on 6C3^+^ CS/DS, further supports the assertion that CS/DS GAG epitopes are likely to be instrumental in cells adopting a less-differentiated phenotype in vitro with the assistance of the CXCR4 receptor.

### 3.4. Serial Passaging and Expression of Indicative Stem Cell Markers 

There is an ongoing effort by researchers to develop culture systems that can optimally regulate stem cell function for the purposes of tissue engineering. Substrates are commonly utilised for this purpose as they have been shown to influence stem cell functions, such as proliferation, induction or differentiation, self-renewal and cellular attachment [60]. Current cell cultivation and expansion procedures used to generate transplantable corneal tissue may take more than three months [61]. Therefore, in the present investigation of the influence of CS/DS-enhanced substrate upon embryonic keratocytes in culture, we extended our observations over multiple passages to determine whether any effects on the expression of cell markers were merely transient or sustained over a longer duration. To our knowledge, the time course of transdifferentiation between phenotypes by embryonic keratocytes is currently unknown, although the assumption is that expression would not change across passages. However, in contrast, the results of this investigation showed there was a significant correlation between the resultant nMedFI and passage for all four indicative stem cell markers when cells were grown on the 6C3^+^ CS/DS coating. Specifically, as cells were repeatedly passaged, Cx43 expression decreased, whereas *PAX6*, Bmi-1 and CXCR4 expression increased. This is suggestive that cells were still in the process of transdifferentiating over the timeframe of the investigation as no reversal or plateau in the expression profile was observed.

### 3.5. Summary

The developing chick has been extensively used as a model system for studies of corneal embryogenesis since the pioneering studies of Hay and Revel [62]. Now, with an increasing wealth of fundamental knowledge about developmental mechanisms, cell-cell and cell-matrix interactions [63,64,65,66,67], the tissue offers many advantages for future studies of stem cells and their niches. Here, we show that keratocytes from late-stage (i.e., E18) embryonic chick corneas show changes in their expression of stem/progenitor cell markers that are suggestive of a less-differentiated, more stem-like phenotype when the cells are cultivated in the presence of 6C3^+^ CS/DS GAGs. These findings are consistent with studies that identify variously sulphated CS/DS epitopes as regulators of stem cell niches in numerous connective and musculoskeletal tissues [13,29,31,36]. It is also noteworthy that the formation of cartilage from bone marrow mesenchymal stem cells (chondrogenesis) is significantly delayed when the CS/DS sulphation motif recognised by 6C3 is disrupted in vitro [68]. In conclusion, data presented here are supportive of a role for 6C3^+^ CS/DS in the retention of a stem/progenitor cell phenotype in keratocytes, with future studies of other CS/DS epitopes, and indeed other GAGs, required to more fully understand the role of these molecules in the stem cell niche of the cornea. These could include in vitro enzyme cleavage of corneal tissue and the GAG preparation with chondroitinase ABC, chondroitinase B and/or chondroitinase C to give support to the specificity of the corneal GAG localization in situ and identify more precisely the GAG coating material and its suggested biological effect, possibly including a structural characterisation of different GAG domains by mass spectrometry glycoproteomics.

## 4. Materials and Methods

### 4.1. Tissue Acquisition

Corneas were dissected from adult Hubbard JA87 chickens obtained freshly slaughtered from a local abattoir (n = 12; Capestone Organic Poultry Ltd., Haverfordwest, UK). Corneas were removed by a parallel incision made approximately 2 mm outside the limbus and fixed in 1% paraformaldehyde in PBS for 1 h, washed and frozen in optimal cutting temperature compound and stored at −80 °C prior to sectioning at 14 µm thickness on a cryostat at −21 °C.

### 4.2. Immunohistochemistry

Sections were washed in PBS containing 0.1% (*v*/*v*) Tween20 before exposure to monoclonal antibody 6C3, which recognises specific native CS/DS sulphation motif epitopes occurring towards the non-reducing terminus. Before treatment with antibody, diluted to 1:20 in PBS/0.1% Tween20, sections were blocked with serum containing PBS/0.1% Tween20 and 1% (*w*/*v*) bovine serum albumin for 30 min. Antibody was applied at 4 °C overnight. To validate the antibody reaction, some sections of E14 tibiotarsal/tarsometatarsal joint were also treated. Following antibody incubation, all sections were washed and then treated with goat anti-mouse AlexaFluor 488 secondary antibody (Invitrogen, Cambridge, UK) diluted to 1:200 in PBS/0.1%Tween20, for 2 h, after which they were washed and then mounted under coverslips with Vectashield containing the nuclear stain 4′,6-diamidino-2-phenylindole (DAPI: Vector Laboratories, Oxford, UK). All sections were examined with an Olympus BX61 fluorescence microscope (Olympus, Stansted, UK). Sections exposed to non-immune serum or PBS instead of the primary antibody served as negative controls. 

### 4.3. Isolation and Culture of Chicken Keratocytes 

Fertilised, white leghorn chicken eggs (Henry Stewart & Co., Ltd., Fakenham, UK) were incubated to E18 before chicks were sacrificed. Corneas (n = 40 ± 2) were dissected and pooled for treatment with 0.04% solution of ethylenediaminetetraacetic acid (Sigma Aldrich, Gillingham, UK) for 45 min at 37 °C incubation. Following incubation, corneas were transferred into a solution of 0.15% collagenase type I (Sigma-Aldrich, Gillingham, UK) that was incubated at 37 °C for 2 h. Resulting supernatant was centrifuged to form a pellet and resuspended in standard culture media containing: Dulbecco’s Modified Eagle Medium (ThermoFisher Scientific, Loughborough, UK), 1% Penicillin/Streptomycin (ThermoFisher Scientific, Loughborough, UK) and 0.25 μg/mL Amphotericin B/Fungizone (ThermoFisher Scientific, Loughborough, UK), and 5% foetal bovine serum (Gibco, ThermoFisher, Loughborough, UK). Cells were seeded onto 35 mm petri dishes at a density of 3.0 × 10^5^ cells/mL and incubated at 37 °C until confluency was reached. Serial passage was performed using dispase II solution (1 U/mL, ThermoFisher Scientific, Loughborough, UK) to dissociate cells.

### 4.4. GAG-Coating of Plates 

The 6C3^+^ CS/DS was extracted from shark skeletal cartilage, a rich source of CS/DS, purified using alkaline beta-elimination, cetyl pyridinium chloride (CPC) precipitation and anion exchange chromatography, as described in Appendix A. The substrate was enriched in CS/DS chains and contained no detectable amounts of highly sulphated keratan sulphate, a common contaminant of CS preparations [69], as ascertained by use of the 5D4 anti-keratan sulphate antibody [70]. For cell culture, plates were pre-coated with 6C3^+^ CS/DS at a density of 0.055 μg/cm^2^ in 80% saturated solution of ammonium sulphate (4.1 M) at 4 °C overnight. Prior to seeding cells, coated surfaces were repeatedly washed with D-PBS. 

### 4.5. Flow Cytometry

At P0 and P2–P5, cells were harvested to form a single-cell suspension at a final concentration of 1 × 10^6^ cells/mL. Samples were subsequently washed with ice cold PBS before resuspension in PBS containing fixable live/dead stain (Live/Dead™ Fixable Far Red Dead Cell Stain Kit, Invitrogen, Cambridge, UK) for 30 min at 4 °C. Following incubation, cells were washed and fixed with 4% PFA in Sorensen’s phosphate buffer (pH 7.4) for 15 min before being washed with PBS. To detect relevant intracellular labelling, applicable samples were permeabilised with 0.2% (*w*/*v*) Tween20 in PBS for 30 min at 4 °C before being washed. 

For immunohistochemical staining, samples were exposed to a panel of primary antibodies: Anti-Connexin 43/GJA1 (Abcam, Cambridge, UK), Anti-PAX6 [AD2.38] (ThermoFisher Scientific, Loughborough, UK), Bmi-1 (Sigma-Aldrich, Gillingham, UK), CXCR4 (Aviva Systems Biology, San Diego, CA, USA), Rabbit IgG- isotype control antibody (Vector Laboratories, Oxford, UK) and Mouse IgG- isotype control antibody (Vector Laboratories, Oxford, UK). Before treatment with antibodies, all of which were diluted to 10 μg/mL in PBS/0.1% BSA, samples were blocked with serum containing PBS/0.1% BSA for 1 h and washed. Antibodies were applied at 4 °C for 1 h and subsequently washed before treatment with secondary antibodies: DyLight 488 Horse Anti-Rabbit and DyLight 488 Horse Anti-Mouse (Vector Laboratories, Oxford, UK) diluted to 1:200 in PBS/0.1% BSA for 45 min. Cells were washed and re-suspended in a final volume of 500 μL of PBS. 

Prior to flow cytometric analysis, visual validation was performed on each sample to assess cellular integrity by pipetting 10 μL of cell suspension onto individual microscope slides. Images were acquired using an Olympus IX71 Inverted Fluorescence and Phase Contrast Tissue Culture Microscope (Olympus, Stansted, UK). 

### 4.6. Data Acquisition

Each sample was analysed on a BD LSRFortessa^TM^ cell analyser (BD, San Diego, CA, USA) equipped with 405/488/561/640 nm lasers. Fluorescein isothiocyanate (FITC) and Allophycocyanin (APC) were detected using the 530/30 and 670/40 filters, respectively. An unstained control was prepared for instrument calibration. A total of 50,000 cells were acquired for each sample. Data were expressed as median fluorescence intensity (MedFI) and a nMedFI, which is the ratio between the MedFI of sample cells and that of the isotypic control cells. A repeat of (n = 5) was performed to establish the consistency of the instrument analysis for each marker, passage and coating condition.

### 4.7. Gating Strategy 

**Figure 4:**
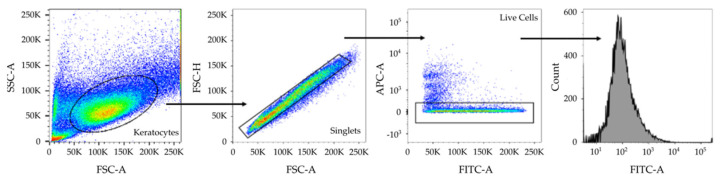
Schematic of sample plots demonstrating the gating strategy employed on raw data in FlowJo to generate graphs. Forward vs. side scatter area (*FSC-A* vs. *SSC-A*) gating was applied to identify cells of interest based on size and complexity (*granularity*), whereas FSC-A vs. forward scatter height (*FSC-H*) gating strategy was applied for the purposes of doublet exclusion. To distinguish between live and dead cells, FSC-A was gated against allophycocyanin area (*APC-A*) to detect fluorescence emitted by the Live/Dead^TM^ Fixable Far Red Dead Cell stain. Finally, a fluorescein isothiocyanate area (*FITC-A*) vs. histogram parameter change was applied to generate a log-distribution of gated events. Pseudocoloured density plots depict the distribution of cells within a population. Blue and green correspond to areas of lower cell density, yellow corresponds to areas of mid-range cell density, and red correspond to areas of high cell density.

Following data acquisition, FC 3.0 files were exported from the BD FACSDiva™ software and imported into FlowJo v.10.6.1 (BD Biosciences, Franklin Lakes, NJ, USA). To the initial graph window of each sample, which displayed the distribution of collected events, gating was applied, as seen in Figure 4, to isolate out positively labelled cells.

### 4.8. Statistics 

Analyses were performed using version 1.2.5003 RStudio software (RStudio Team, Boston, MA, USA). To investigate whether the expression of indicative stromal stem cell markers Cx43, *PAX6*, Bmi-1 and CXCR4 were affected by coating condition, a paired student *t*-test was performed. *p*-values less than 0.05 were considered significant. The mean of the differences and associated 95% confidence intervals are also reported throughout the analysis. To investigate the relationship between nMedFI and passage number for each indicative stromal stem cell marker and coating condition, repeat measurements of nMedFI were taken using flow cytometry. Linear regression analyses (model I; ‘lm’ package [71]) were then used to fit regression lines between measurements of nMedFI and passage number for each coating condition. Figures showing linear regression were generated using the ggplot2 package in RStudio.

## Figures and Tables

**Table 1:** Summary of indicative stem cell marker expression (nMedFI) across passages for E18 keratocytes cultured on uncoated and 6C3^+^ CS/DS coated culture plates.

Marker	P0	P2	P3	P4	P5	Average (Mean nMedFI ± SD)
n	5	5	5	5	5	
Uncoated (Mean nMedFI) *						
Cx43	0.77	1.46	1.35	1.49	1.67	1.35 ± 0.34
PAX6	0.14	0.18	0.12	0.11	0.13	0.14 ± 0.03
Bmi-1	1.31	1.79	0.97	0.79	1.75	1.32 ± 0.45
CXCR4	0.29	0.38	1.67	1.62	1.55	1.10 ± 0.70
6C3^+^ CS/DS Coated (Mean nMedFI) *						
Cx43	0.77	0.23	0.19	0.19	0.18	0.31 ± 0.26
PAX6	0.14	0.34	0.27	0.40	0.45	0.32 ± 0.12
Bmi-1	1.31	1.74	2.39	1.53	2.65	1.92 ± 0.57
CXCR4	0.29	3.36	3.70	2.37	3.58	2.66 ± 1.42

* The variability between repeated analyses of each sample for all passages and coating conditions never exceeded 14.6%.

**Table 2:** Paired student *t*-test of nMedFI for each indicative stem cell marker and coating condition.

Marker	Paired *t*-Test
Cx43	Mean Difference 1.04, 95% CI [0.81, 1.26], *p* < 0.001
PAX6	Mean Difference −0.18, 95% CI [0.23, 0.14], *p* < 0.001
Bmi-1	Mean Difference −0.60, 95% CI [0.83, 0.37], *p* < 0.001
CXCR4	Mean Difference −1.56, 95% CI [2.00, 1.12], *p* < 0.001

**Table 3:** Regression coefficients for the relationship between nMedFI and passages for each coating condition and indicative stem cell marker.

Marker	Statistic	Uncoated	6C3^+^ CS/DS Coated
Cx43	r	0.18	−0.12
	R^2^	0.70	0.54
	*p*	<0.001	<0.001
PAX6	r	−0.01	0.06
	R^2^	0.30	0.78
	*p*	0.002	<0.001
Bmi-1	r	−0.01	0.25
	R^2^	0.04	0.45
	*p*	0.86	<0.001
CXCR4	r	0.38	0.56
	R^2^	0.70	0.36
	*p*	<0.001	<0.001

## Data Availability

Data will be made available on reasonable request.

## Appendix A. Method Used to Extract and Purify 6C3^+^ CS/DS

The following method outlines how 6C3^+^ CS/DS GAGs were extracted and purified from shark skeletal cartilage for use as substrates for chick corneal keratocyte culture.

### Appendix A.1. Proteoglycan Extraction

An amount of 10 mL of 4 M guanidine hydrochloride at pH 5.8 per gram of tissue (wet weight) was added to minced shark skeletal cartilage along with 0.05 M sodium acetate and 10 mM sodium ethylene-diamine-tetraacetate (Na2EDTA) in the presence of the following protease inhibitors: 0.1 M 6-amino-caproic acid and 5 mM benzamidine hydrochloride. The tissue was incubated in extraction buffer for 48 h at 4 °C on a roller mixer. After incubation in 4 M guanidine hydrochloride, tissue was centrifuged at 3000× *g* for ten minutes and the supernatant removed and passed through a 40 μm cell strainer (ThermoFisher Scientific, Loughborough, UK) to remove debris resulting in a crude extract. This was subsequently dialysed to the same solution as above, but with 0.4 M guanidine hydrochloride prior to associative isopycnic density ultracentrifugation.

### Appendix A.2. Density Gradient Centrifugation

Caesium chloride was added to the crude proteoglycan supernatant to achieve a density of 1.5 g/mL (approximately 0.615 g/mL of caesium chloride added), after degassing using a vacuum pump and maintaining the guanidine hydrochloride molarity at 4 M for associative isopycnic ultracentrifugation. Samples were subsequently ultracentrifuged at 100,000× *g* for 48 h separating the sample mixtures based on molecular density until equilibrium is reached. Density ultracentrifugation was performed using a Beckman Coulter L8-80MR Ultracentrifuge fitted with a 55.2 Ti rotor in 26.3 mL polycarbonate ultracentrifuge tubes (all from Beckman Coulter, High Wycombe, UK). Three associative fractions were generated. These fractions are termed A1 to A3 (A1 being the highest buoyant density and A3 being the lowest buoyant density, i.e., at the top of the density gradient). The gradient densities of each fraction were verified by weighing each fraction. Samples were then dialysed into distilled water following several water changes maintained at 4 °C over 48 h and used in further analysis or frozen at −20 °C in aliquots for further use. Unless otherwise stated, 500 µg of proteoglycan fractions from density centrifugation were freeze-dried and reconstituted in 1 mL of distilled water for alkaline β-elimination of samples.

### Appendix A.3. Alkaline β-Elimination

Alkaline β-elimination is well described, and releases O’ linked glycans from proteins in solution through an elimination reaction [72] and can be performed in the presence of sodium borohydride or without [73]. Initially, an equal volume of 1 M NaOH was added to the prepared sample fractions in distilled water, yielding a 0.5 M solution of NaOH that was incubated overnight at room temperature. An equal volume of 1 M acetic acid was then added to neutralise the solution and the sample was then dialysed extensively against distilled water. Samples were then freeze-dried and reconstituted in 0.5 mL of 50 mM sodium sulphate.

### Appendix A.4. CPC Precipitation

CPC has a high affinity for GAGs and can be used to selectively precipitate them, and accordingly is a suitable molecule for a washing step prior to anion exchange chromatography [74]. After β-elimination, CPC solution was added dropwise to samples in 50 mM sodium sulphate (10% (*w*/*v*)) at room temperature until a dense, cloudy precipitate formed, which was the resultant CPC salt. The samples were then centrifuged at room temperature for 5 min at 2000× *g* and the supernatant discarded. The pellet was then washed twice in 0.05% (*w*/*v*) CPC to remove further contaminants. After the wash step, 0.5 mL of 80% (*v*/*v*) propan-1-ol in distilled water was added, in which the CPC precipitate dissolves, with the resultant jelly-like product mixed to form a solution. To this solution, 100 µL of saturated sodium acetate solution was then added along with a drop of acetic acid to decrease the pH of the solution. This resulted in the formation of a glycosaminoglycan sodium salt. Finally, 3 mL of cold ethanol was added to precipitate the sodium salt of the glycosaminoglycan chains as a dense white precipitate, which was achieved overnight at 4 °C on a roller mixer. Samples were then centrifuged, again at 2000× *g*, the supernatant discarded, and the samples dried before being reconstituted to 1 mL in anion exchange starting buffer (0.1 M NaCl in 10 mM Tris-HCl at pH 7) and applied to an anion exchange column as described below.

### Appendix A.5. Anion Exchange Chromatography

In this study the reaction product from the ß-elimination and CPC washing of free O’ linked glycosaminoglycan side chain mixtures was applied to a 5 mL HiTrap Q Fast Flow column (Cytiva Life Sciences, Sheffield, UK), attached to an ÄKTA explorer P-900 anion exchange chromatography system (GE Healthcare, Amersham, UK). The run conditions were as follows and have been described previously [69]. Samples were run at a flow rate of 2 mL/min on a linear buffer gradient from 0.1 M NaCl to 3 M NaCl in 10 mM Tris-HCl at pH 7.4 (after filtering buffer using a 0.22 µm filter) for eight column volumes (40 mL total). The column was equilibrated with 5 column volumes of starting buffer before application of the proteoglycan or GAG sample. Overall, 16 fractions were collected of 2.5 mL each. UV absorbance at 232 nm was monitored for detection of GAGs. Fractions that eluted during peak 232 nm UV absorbance were deemed of interest and these resultant fractions were dialysed into distilled water and measured for GAG and protein content.

### Appendix A.6. Direct ELISA

To detect GAG sulphation motifs present in the generated samples, direct ELISA was performed. Anion exchange chromatography fractions, with a notable UV absorbance at 232 nm and detectable GAG content (ascertained from the DMMB assay) were coated onto Linbro 96 microwell ELISA plates (mpbio/ThermoFisher Scientific, Loughborough, UK). The free GAGs were coated using 100 μL of 1 μg/mL GAG solution (again, determined by the DMMB assay) using 80% saturated ammonium sulphate (4.1 M) as the coating buffer, a technique that has previously been used for optimal immobilisation of free CS/DS GAG chains [75]. The samples were then covered and incubated at 4 °C for 16 h, after which the ELISA was performed as below. All samples were performed in duplicate and included a negative control of primary antibody omission. Following incubation, the plates were washed twice with TSA wash buffer (as above but with 0.05% Tween). The plates were then blocked with 5% (*w*/*v*) BSA/TSA wash buffer for 1 h at 37 °C before addition of the primary antibodies. Primary antibodies, 6C3 (CS/DS sulphation motif, 1:10), 5D4 (keratan sulphate sulphation motif 1:100) and 7D4 (CS/DS sulfation motif, 1:10) were all diluted in 1% BSA/TSA wash buffer and applied to the plate undergoing incubation for 1 h at 37 °C. After the primary antibody incubation, microplates were washed, and the goat anti-mouse alkaline phosphatase conjugated secondary antibody (ThermoFisher Scientific, Loughborough, UK) was applied at a 1 in 5000 dilution in 1% BSA/TSA wash buffer, as per manufacturer’s instructions, and incubated for 1 h at 37 °C. The plates were then thoroughly washed. Finally, samples were incubated with alkaline phosphatase substrate (4-nitrophenyl phosphate disodium salt hexahydrate) (Sigma-Aldrich, Gillingham, UK), dissolved in diethanolamine (DEA) buffer (5 mg alkaline phosphatase substrate per 5 mL 0.1 M DEA buffer with 1 mM MgCl2 at pH 9.8) for 1 h at 37 °C, after which the yellow reaction product absorbance was read at 405 nm on a CLARIOstar plate reader (BMG Labtech, Aylesbury, UK). This analysis confirmed that the 6C3^+^ CS/DS shark skeletal cartilage extract used to coat plates for chick keratocyte culture contained no detectable keratan sulphate or protein contaminants.
